# Systematic Quantification of Protein O‑GlcNAcylation Reveals Common and Cell-Type-Specific Responses to N‑Glycosylation Inhibition in Human Cells

**DOI:** 10.1021/acs.analchem.6c00972

**Published:** 2026-05-19

**Authors:** Longping Fu, Kejun Yin, Xing Xu, Ronghu Wu

**Affiliations:** School of Chemistry and Biochemistry and the Petit Institute for Bioengineering and Bioscience, 1372 Georgia Institute of Technology, Atlanta, Georgia 30332, United States

## Abstract

Both protein O-GlcNAcylation and N-glycosylation are extremely important in human cells and regulate many cellular events. While O-GlcNAcylation is known to act as a stress sensor, its changes in human cells with N-glycosylation perturbations remain to be explored. In this study, we comprehensively and site-specifically studied common and cell-type-specific responses of protein O-GlcNAcylation under N-glycosylation inhibition in three types of human cells (HEK293T, HepG2, and Jurkat cells) by integrating metabolic labeling, bio-orthogonal chemistry, and multiplexed proteomics. In total, more than 1000 O-GlcNAcylated proteins were identified and quantified, and the results demonstrate that under the inhibition of protein N-glycosylation, O-GlcNAcylated proteins related to stress response and translation are commonly changed in different types of cells. Furthermore, O-GlcNAcylation changes are cell-type-specific, and O-GlcNAcylated proteins related to leukocyte proliferation and T-cell activation were upregulated in Jurkat cells, while in HEK293T cells, those associated with ribonucleotide metabolism and ribosome biogenesis were upregulated. Site-specific analysis revealed that O-GlcNAcylation sites in structured regions exhibited larger abundance changes compared with those in intrinsically disordered regions. This study provides valuable insights into the regulation of protein O-GlcNAcylation in human cells under N-glycosylation inhibition, advancing our understanding of protein glycosylation.

## Graphical abstract

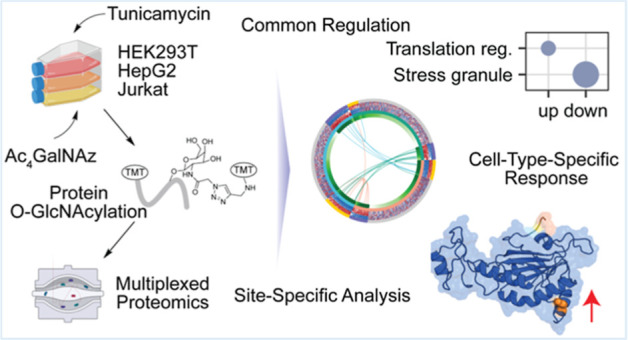

Glycosylation is one of the most important and common protein modifications, and it plays vital roles in regulating protein activities and many cellular events. Unlike modifications with a single defined moiety, such as phosphorylation and acetylation, protein glycosylation encompasses structurally diverse modification types, including N-glycosylation, mucin-type O-glycosylation, and O-GlcNAcylation. Protein O-GlcNAcylation is a dynamic and reversible modification where a single monosaccharide, i.e., *N*-acetylglucosamine (GlcNAc), is bound to the serine, threonine, or tyrosine residue, predominantly on nuclear and cytoplasmic proteins.^1–4^ It regulates various cellular events, including gene expression and signal transduction.^5^ Additionally, dysregulation of protein O-GlcNAcylation has been implicated in human diseases, such as neurodegenerative diseases and diabetes.^6,7^ In contrast, protein N-glycosylation involves attachment of a preassembled core glycan to asparagine (Asn) residues in the ER; this glycan is further elaborated in the ER and the Golgi apparatus, contributing to protein folding, trafficking, and regulation of many extracellular events.^8^

While protein O-GlcNAcylation and N-glycosylation differ in their glycan structures and functions, they also share some similarities. For example, uridine diphosphate *N*-acetylglucosamine (UDP-GlcNAc) is used to synthesize the core *N*-glycan (GlcNAc_2_Man_9_Glc_3_) and also serves as the sugar donor for protein O-GlcNAcylation.^9^ Furthermore, protein O-GlcNAcylation has been reported to modulate protein solubility, especially in pathological contexts such as preventing protein tau from its toxic self-assembly.^10^ Similarly, N-glycosylation sites are enriched near aggregation-prone regions and appear to be evolutionarily selected, especially in proteins prone to misfolding.^11^ The established role of protein O-GlcNAcylation in cellular stress responses suggests potential crosstalk between protein O-GlcNAcylation and N-glycosylation because the N-glycosylation inhibition induces ER stress. Additionally, a negative relationship was reported between the levels of protein O-GlcNAcylation and N-glycosylation in brain and serum samples from patients with Alzheimer’s disease.^12^ However, comprehensive and quantitative analysis of protein O-GlcNAcylation under N-glycosylation perturbation across different cell types has yet to be reported.

In this work, we systematically and site-specifically analyzed protein O-GlcNAcylation in human cells under N-glycosylation inhibition through the integration of metabolic labeling, bio-orthogonal chemistry, and multiplexed proteomics. The experiments were performed in three types of human cells, i.e., HEK293T, HepG2, and Jurkat cells. Under the inhibition of protein N-glycosylation in cells using tunicamycin (Tm), we systematically studied protein O-GlcNAcylation, and a total of 1,109 O-GlcNAcylated proteins were characterized. The current results revealed distinct cell-type-specific responses to the inhibition of protein N-glycosylation. At the same time, it was found that commonly upregulated O-GlcNAcylated proteins were enriched in translation regulation and glucose response, whereas commonly downregulated proteins were associated with stress granule assembly and stress response regulation. It was found that local structures of O-GlcNAcylation sites are a key determinant of their changes. O-GlcNAcylation sites in structured regions show significantly higher abundance changes compared with those in intrinsically disordered regions (IDRs), where O-GlcNAcylation sites remained largely unaffected. This study advances our understanding of the properties and functions of protein glycosylation.

## Experimental Section

### Cell Culture and Metabolic Labeling

HEK293T and HepG2 cells were cultured in Dulbecco’s Modified Eagle Medium (DMEM, Sigma-Aldrich) containing 10% fetal bovine serum (FBS, Phoenix Scientific) and 1% penicillin–streptomycin (Sigma-Aldrich). Jurkat cells were cultured in Roswell Park Memorial Institute (RPMI) 1640 medium (Sigma-Aldrich) containing 10% FBS and 1% penicillin–streptomycin. All cells were grown in a humidified incubator with 5.0% CO_2_ at 37 °C. When HEK293T and HepG2 cells reached ∼50% confluency, they were treated with Tm (2 μg/mL, MedChemExpress) or DMSO (0.1%, Sigma-Aldrich) as a control, respectively. When the density of Jurkat cells reached 5 × 10^5^ cells/mL, they were treated similarly. After the treatment for 24 h, cells were supplemented with 200 μM *N*-azidoacetylgalactosamine-tetraacylated (Ac_4_GalNAz, Vector Laboratories) along with Tm and were further treated for another 24 h.

### Click Chemistry and O-GlcNAcylated Peptide Enrichment

For glycoprotein analysis, labeled glycoproteins were subjected to the copper­(I)-catalyzed azide–alkyne cycloaddition (CuAAC) reaction. For glycopeptide enrichment, purified peptides were incubated with Pierce High Capacity NeutrAvidin Agarose resins (Thermo Scientific) according to the manufacturer’s protocol with some modifications. The eluents were combined, desalted, lyophilized, and stored at −80 °C. (Detailed information is available in the Supporting Information.)

### Sample Fractionation Using HPLC and LC-MS/MS Analysis

The TMT-labeled peptides were separated by high-pH HPLC into 12 fractions. The purified samples were dissolved in 6 μL of 0.1% formic acid (FA, Sigma-Aldrich), and 4 μL was loaded onto a microcapillary column packed with C18 beads (ReproSil-Pur 120 C18-AQ, 1.9 μm, Dr. Maisch). Peptides were separated using a Vanquish Neo UHPLC system (Thermo Scientific) and analyzed by an Orbitrap Eclipse Tribrid Mass Spectrometer (Thermo Scientific). (More information such as database search, data filtering, and data analysis is available in the Supporting Information.)

### Quantification and Statistical Analysis

Glycopeptides and glycoproteins that exhibited |log_2_(Tuni/Ctrl)| >0.5 and adjusted *P* value (Benjamini–Hochberg) <0.05 were considered significantly regulated (more information is in the Supporting Information).

## Results

### Principle of Comprehensively Studying Protein O-GlcNAcylation under N-Glycosylation Inhibition

To investigate the responses of protein O-GlcNAcylation under N-glycosylation perturbation, we inhibited protein N-glycosylation using Tm, an antibiotic that specifically blocks the first step of protein N-glycosylation by inhibiting *N*-acetylglucosamine-1-phosphate transferase.^13,14^ Then, O-GlcNAcylated proteins were comprehensively quantified in a site-specific manner by mass spectrometry (MS), as shown in Figure 1. Modern MS-based proteomics provides a unique opportunity to globally and site-specifically analyze protein modifications,^15–18^ including glycosylation.^19–27^ The concentration of Tm was chosen based on the previous reports.^28–30^ Cells were treated with Tm for 24 h and then supplemented with the sugar analog (Ac_4_GalNAz, 200 μM) for another 24 h to label O-GlcNAcylated proteins together with Tm. O-GlcNAcylated proteins metabolically labeled with the azido group were subsequently tagged with Photocleavable (PC) Biotin Alkyne. After tryptic digestion, glycopeptides were captured using NeutrAvidin agarose resins and were sequentially released under the radiation (365 nm).

**Figure 1:**
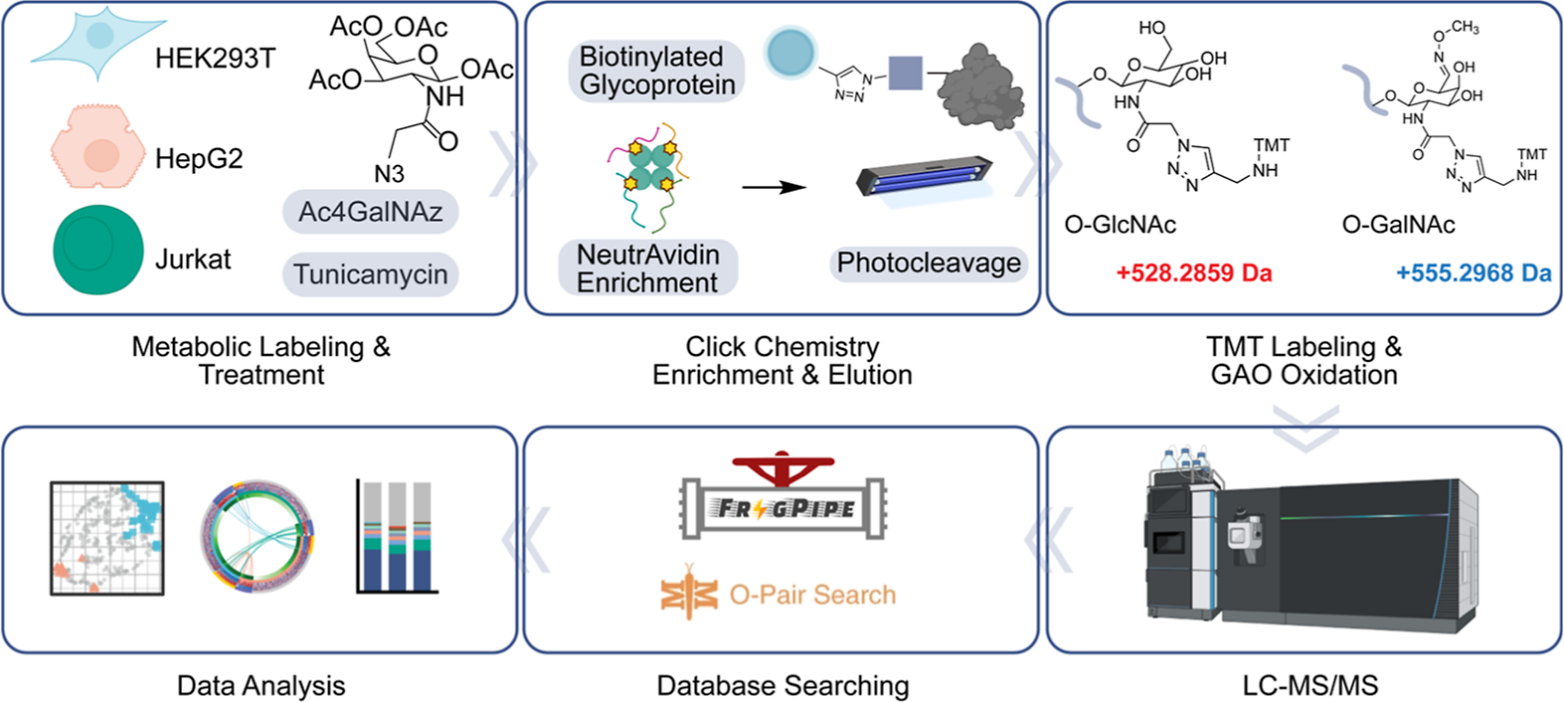
Experimental workflow for quantifying protein O-GlcNAcylation in HEK293T, HepG2, and Jurkat cells with the inhibition of protein N-glycosylation.

Ac_4_GalNAz can be incorporated into both O-GlcNAc and O-GalNAc (Tn antigen) modifications, which cannot be readily distinguished by MS because they have the same chemical compositions and masses. As we did previously, benefiting from the specificity of an enzyme reaction, galactose oxidase (GAO) was used to distinguish them, and then they have different tags for MS analysis.^31^ In brief, the enriched glycopeptide samples were treated with GAO, which oxidizes the Tn antigen, but not O-GlcNAc, followed by methoxylamine labeling to selectively derivatize O-GalNAcylated peptides. Therefore, the mass tags on the two modifications are dramatically different (528.2859 vs 555.2968, Figure 1). This mass shift of 27 Da allows us to clearly distinguish and confidently analyze O-GlcNAcylated and O-GalNAcylated proteins by MS.

On combining selective enrichment with multiplexed proteomics, we can globally quantify O-GlcNAcylated proteins. Glycopeptides were analyzed by LC-MS/MS using a higher-energy collisional dissociation product-dependent-electron-transfer/higher-energy collisional dissociation (HCD-pd-EThcD) fragmentation strategy, where HCD is for glycopeptide identification, while product-dependent EThcD preserves the labile O-glycosidic bond and produces *c*/*z* ions for confident localization of O-GlcNAcylation sites. Peptide identification and O-glycosylation site localization were performed using FragPipe with O-Pair Search.^32,33^ To quantify the changes of O-GlcNAcylated proteins when N-glycosylation was inhibited, we calculated the log_2_(fold change) (log_2_ FC, log_2_(Tuni/Ctrl)) for each glycopeptide and glycoprotein based on the TMT reporter ion intensities. The reproducibility across the biological triplicate experiments was also evaluated and found to be reasonably high in each type of cells (Figure S1).

### Global Identification of Protein O-GlcNAcylation in Human Cells with N-Glycosylation Inhibition

After effective enrichment and distinguishing glycoproteins with O-GlcNAc and O-GalNAc, a total of 1,109 O-GlcNAcylated proteins were identified across HEK293T, HepG2, and Jurkat cells (Figure 2; Table S1). We identified 775 glycoproteins in HEK293T cells, 676 in HepG2 cells, and 692 in Jurkat cells with 402 O-GlcNAcylated proteins commonly identified in all three cell types. GO enrichment analysis revealed that among commonly identified glycoproteins, those related to transcription coregulator activity, mRNA binding, nucleocytoplasmic transport, nuclear transport, histone acetyltransferase activity, and cytoplasmic stress granules are significantly enriched (Figure 2). This is consistent with the well-documented functions of protein O-GlcNAcylation. Cell-type-specific functions of O-GlcNAcylated proteins were also examined. Among glycoproteins exclusively identified in HEK293T cells, the overrepresented GO terms include chaperone-mediated folding, the cell cycle process, and epithelial cell proliferation. These are consistent with the epithelial morphology and high proliferative capacity, which are characteristic of this cell type. O-GlcNAcylated proteins only identified in Jurkat cells were enriched in terms related to leukocyte activation, leukocyte adhesion, and T-cell activation, reflecting the immunological functions of this T lymphocyte cell type. These results demonstrate both common and cell type-specific functions of O-GlcNAcylated proteins, highlighting their functional diversity across different biological contexts.

**Figure 2:**
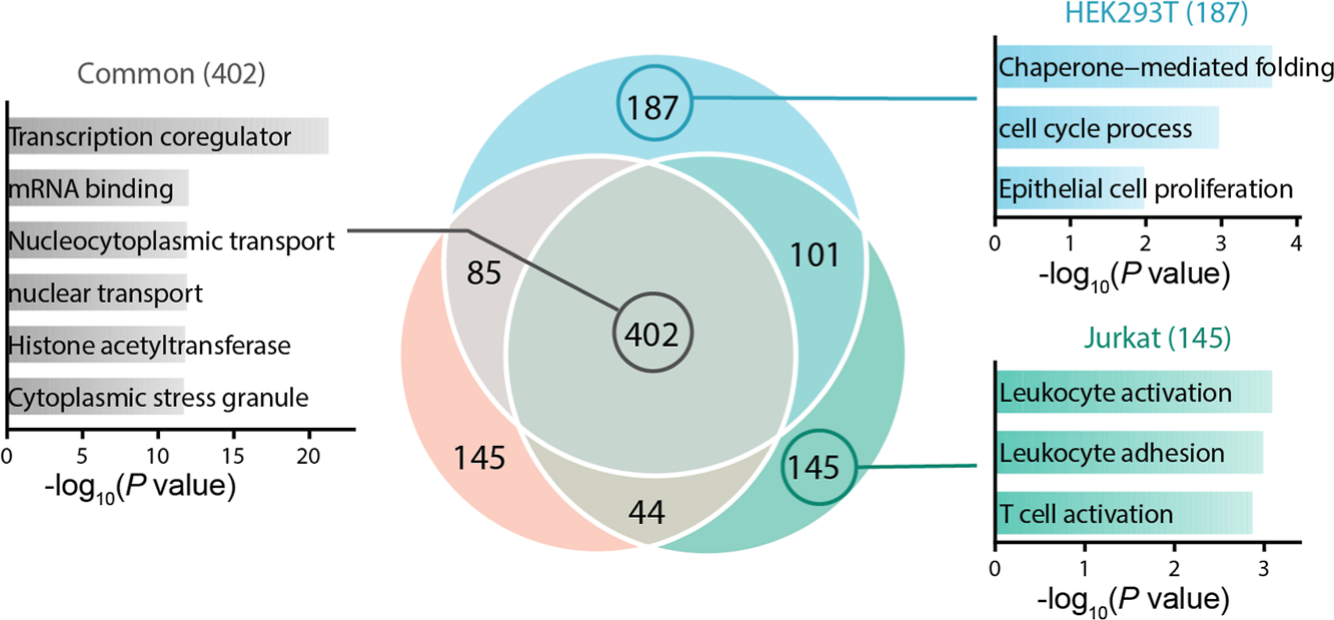
Identification of O-GlcNAcylated proteins in HEK293T, HepG2, and Jurkat cells. Overlap of identified O-GlcNAcylated proteins across three cell types. GO enrichment analysis of O-GlcNAcylated proteins commonly identified across all three cell types or exclusively in HEK293T or Jurkat cells.

### Systematic Quantification of O-GlcNAcylated Proteins and Common Changes of O-GlcNAcylated Proteins across the Cell Types under N-Glycosylation Inhibition

Using TMT-based multiplexed proteomics, we systematically quantified O-GlcNAcylated proteins in each type of cells with the inhibition of N-glycosylation (Figure 3, Table S2). The distribution of log_2_FC values for O-GlcNAcylated proteins showed significant differences among cell types (Figure 3A), indicating cell-type-specific responses of protein O-GlcNAcylation to the perturbation of N-glycosylation. When comparing the distribution of the log_2_(Tuni/Ctrl) values of O-GlcNAcylated proteins with their nonmodified counterparts, dramatic differences were observed among these three cell types (Figure 3B, Tables S3 and S4). This result clearly indicates that protein O-GlcNAcylation is more dynamic compared to their nonmodified forms in cells with the N-glycosylation inhibition.

**Figure 3:**
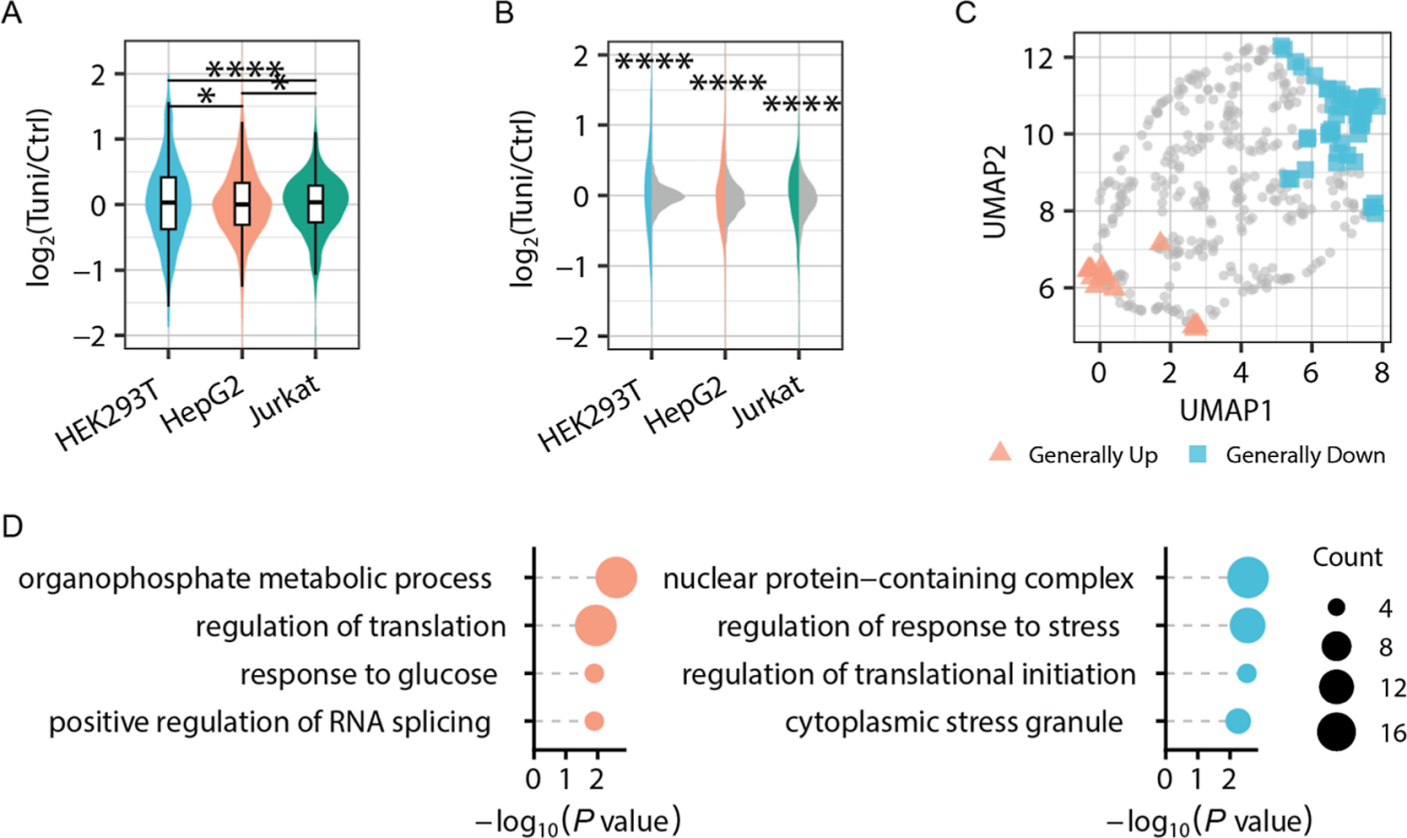
Quantitative analysis of protein O-GlcNAcylation across different cell types under N-glycosylation inhibition. (A) Distribution of log_2_(Tuni/Ctrl) values of O-GlcNAcylated proteins across cell types, and statistical comparisons were performed between cell types. (B) Comparison of abundance changes between O-GlcNAcylated proteins (colored) and their nonmodified counterparts (gray) in each cell type. (C) UMAP analysis of generally up- and downregulated O-GlcNAcylated proteins across cell types. (D) Overrepresented GO terms among generally upregulated (left) or downregulated (right) O-GlcNAcylated proteins. Statistical significance was determined by the Kolmogorov–Smirnov (K–S) test. The significance levels are represented as ****P value <0.0001, ***P value <0.001, **P value <0.01, and *P value <0.05.

To understand the general changes of O-GlcNAcylated proteins in different types of cells with the N-glycosylation inhibition, we performed an analysis for those up- or downregulated glycoproteins in different types of cells. O-GlcNAcylated proteins that are significantly upregulated in at least two types of cells and not downregulated in the other one are classified as commonly upregulated glycoproteins, while those significantly downregulated in at least two types of cells and not upregulated in the other one are commonly downregulated ones. UMAP analysis was performed to visualize the distribution of these generally regulated O-GlcNAcylated proteins (Figure 3C). Among the generally upregulated O-GlcNAcylated proteins, those related to the organophosphate metabolic process, regulation of translation, response to glucose, and positive regulation of RNA splicing were enriched (Figure 3D). Among the generally downregulated glycoproteins, the GO terms of nuclear protein-containing complexes, regulation of response to stress, regulation of translational initiation, and cytoplasmic stress granules were enriched. The bidirectional regulation of translation-related processes is consistent with the dual effect of the integrated stress response.^34,35^ Prior studies have implicated protein O-GlcNAcylation in the dynamics of biomolecular condensates, modulating their assembly and disassembly under cellular stress conditions.^36,37^ Our results further support the role of protein O-GlcNAcylation as a cellular stress sensor in these processes.

### Cell-Type-Specific Changes of O-GlcNAcylated Proteins under N-Glycosylation Inhibition

To further explore the cell-type-specific changes of protein O-GlcNAcylation with N-glycosylation perturbation, we analyzed regulated glycoproteins in each cell type. We found that 163, 113, and 62 glycoproteins were upregulated, and 146, 96, and 86 glycoproteins were downregulated in HEK293T, HepG2, and Jurkat cells, respectively (Figure 4A, Table S2). In Jurkat cells, proteins with upregulated O-GlcNAcylation are enriched for the regulation of leukocyte proliferation, leukocyte migration, cell activation, lymphocyte activation, and regulation of T-cell activation (Figure 4B). These enrichment patterns directly reflect the T lymphocyte identity of Jurkat cells. O-GlcNAcylation cycling is essential for T-cell progenitor renewal, clonal expansion, and malignant transformation, as the loss of OGT prevents these processes and blocked O-GlcNAc cycling disrupts early T-cell specification and thymocyte development.^38,39^ The increased level of O-GlcNAcylation of proteins involved in leukocyte proliferation and activation under the N-glycosylation inhibition may represent an adaptive response to maintain T-cell function during cellular stress.

**Figure 4:**
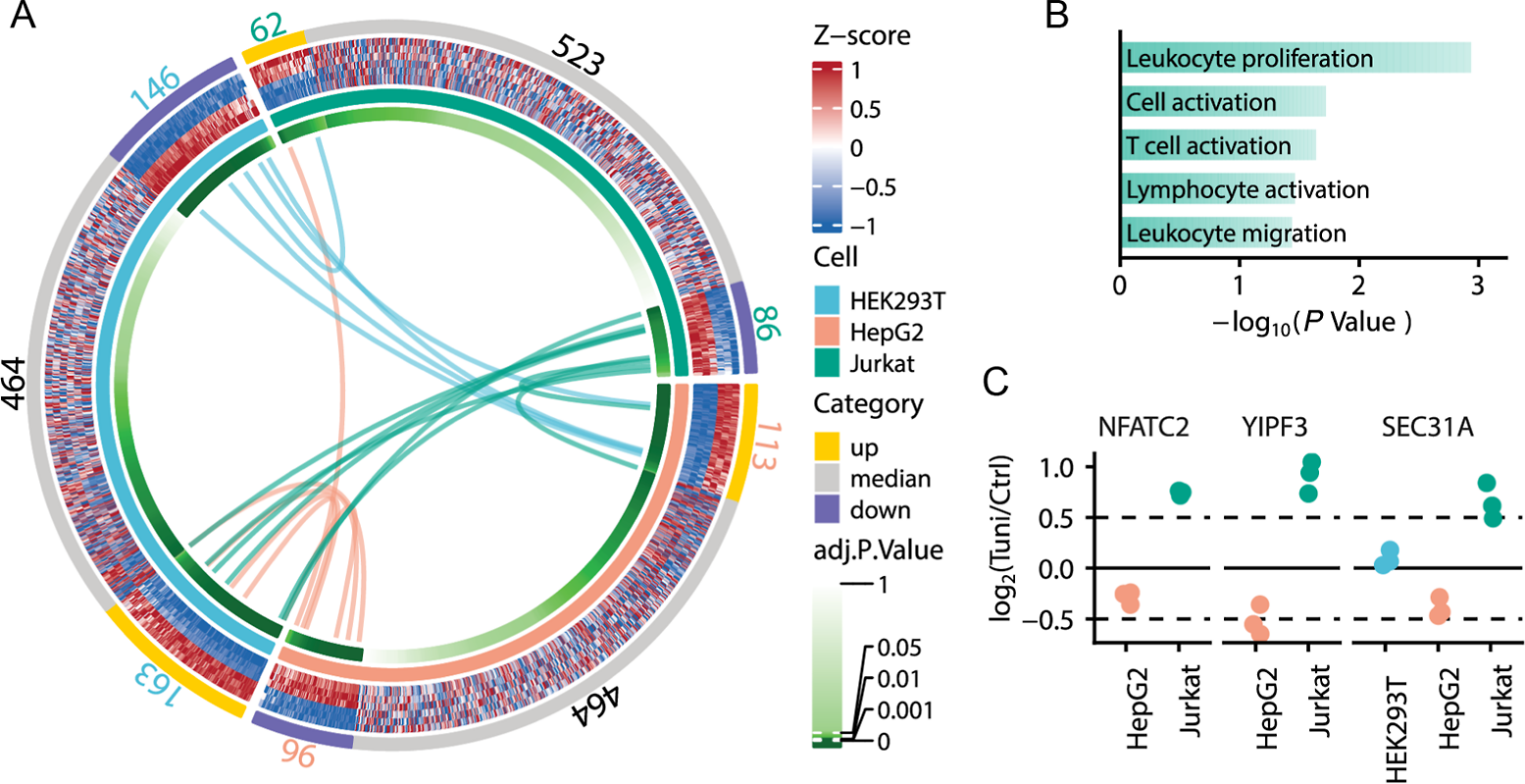
Investigation of significantly regulated O-GlcNAcylated proteins in HEK293T, HepG2, and Jurkat cells. (A) Circular heatmap of quantified glycoproteins across three types of cells. Links within the heatmap highlight instances where the same glycoproteins are differentially regulated among the cell types. (B) GO terms enriched in glycoproteins upregulated only in Jurkat cells. (C) Selected examples of O-GlcNAcylated proteins differentially regulated among cell types. Dashed lines indicate the fold change threshold.

Some examples of O-GlcNAcylated proteins that were differentially regulated in Jurkat cells are shown in Figure 4C. NFATC2, a calcium-regulated transcription factor important for T-cell activation, showed upregulation in Jurkat cells but downregulation in HepG2 cells.^40,41^ YIPF3, a Golgi-associated transmembrane protein involved in Golgi maintenance, was upregulated in Jurkat cells but downregulated in HepG2 cells. Furthermore, SEC31A, a COPII coat component essential for ER-to-Golgi vesicle transport, also exhibited divergent regulation—downregulated in HepG2 cells but upregulated in Jurkat cells, suggesting that O-GlcNAcylation of secretory pathway components may be modulated through distinct, cell-type-dependent mechanisms.^42^ This pattern is consistent with the enrichment of leukocyte activation pathways and vesicle transport observed in Jurkat cells.

Among proteins with upregulated O-GlcNAcylation unique to HEK293T cells, those related to ribonucleotide metabolic process, ribosome biogenesis, response to retinoic acid, forebrain generation of neurons, and protein folding are highly overrepresented (Figure S2). The enrichment of ribonucleotide metabolism and ribosome biogenesis is consistent with the high proliferative capacity and biosynthetic demand of this cell line. Notably, the enrichment of retinoic acid response and neuronal generation terms aligns with the neuronal characteristics of HEK293 cells, which express multiple neuron-specific proteins, despite their embryonic kidney origin.^43^ The enrichment of protein folding is consistent with the high biosynthetic activity of HEK293T cells and may represent an adaptive response to maintaining proteostasis during tunicamycin-induced ER stress. Together, these results demonstrate that O-GlcNAcylated proteins exhibit cell-type-specific responses to N-glycosylation inhibition, reflecting the distinct functional characteristics of each cell type.

### Site-Specific Analysis of Protein O-GlcNAcylation in Response to the Inhibition of N-Glycosylation

Besides protein-level quantification, site-specific analysis can provide unique information about the sequence and structural context of individual O-GlcNAcylation sites influencing their responses to the N-glycosylation inhibition. To ensure high confidence in site assignments, we applied stringent filtering criteria prior to site-level analysis. Previous studies have demonstrated that per-O-acetylated sugar analogs can induce nonenzymatic S-glycosylation on cysteine residues, which may interfere with O-GlcNAcylation identification.^44,45^ To avoid the interference of S-GlcNAcylation, we applied the following stringent criteria outlined previously.^46^ Any glycopeptides with glycan localized on the cysteine residue (confidence level 1 or level 1b) were removed. Furthermore, any identified glycopeptides with a cysteine residue in their sequences with confidence level 2 or level 3 were also deleted. Eventually, only confidently localized glycopeptides (confidence level 1 or level 1b) with the glycan on the serine, threonine, or tyrosine residue were used for site-specific analysis (Table S5). Additionally, a site probability threshold of ≥0.75 was applied to all glycopeptide-spectrum matches to ensure confident site localization. The O-GlcNAcylation site information should be of high confidence after combining all these factors: distinguishing glycopeptides with O-GlcNAc and O-GalNAc benefiting from the specificity of the enzymatic reaction, using the HCD-pd-EThcD fragmentation method, removing glycopeptides with potential modification on cysteine, and including only confidently localized glycopeptides (confidence level 1 or level 1b) with the glycan on the serine, threonine, or tyrosine residue.

Because the changes in O-GlcNAcylation sites can vary in response to the inhibition of N-glycosylation, we examined the local environment surrounding these sites. For site quantification, besides that the sites are well-localized using the stringent criteria described above, only singly glycosylated peptides are considered. The distributions of fold changes of O-GlcNAcylation sites differed among the three cell types (Figure 5A and Table S6). Additionally, the majority of quantified O-GlcNAcylation sites were located in unstructured regions, followed by bend and strand conformations (Figure 5B), consistent with previous reports that O-GlcNAcylation preferentially occurs in disordered regions.^47^

**Figure 5:**
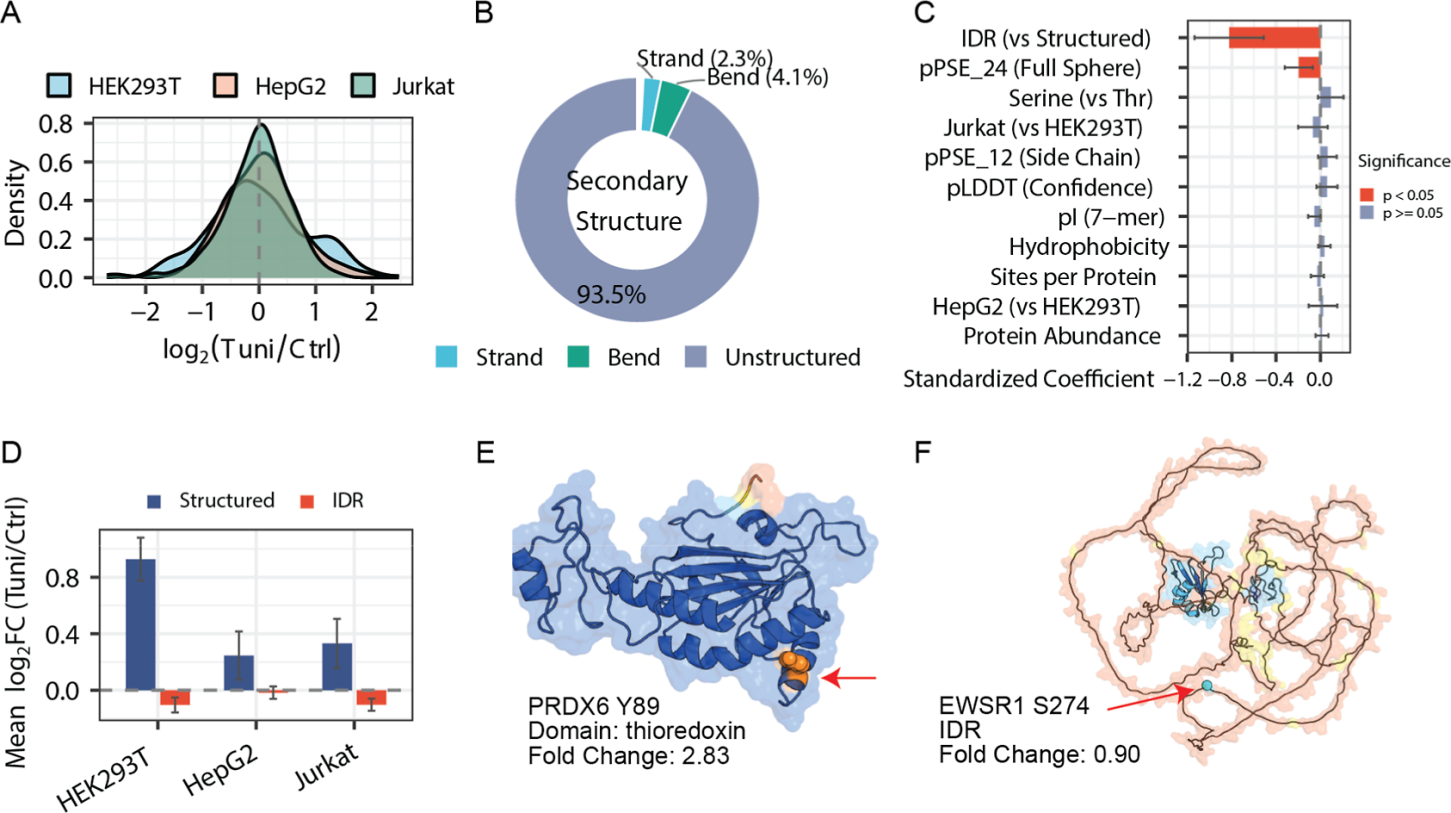
Analysis of protein O-GlcNAcylation sites in response to the inhibition of N-glycosylation. (A) Distributions of fold changes of O-GlcNAcylation sites across three cell types. (B) Proportion of O-GlcNAcylation sites in different secondary structures. (C) Standardized regression coefficients from multiple linear regression models predicting site-level fold changes. Error bars represent 95% confidence intervals. (D) Effect of intrinsically disordered regions (IDRs) on the abundance changes of O-GlcNAcylation sites across cell types. Error bars represent one standard deviation. (E) AlphaFold-predicted structure of PRDX6 colored by pLDDT (predicted Local Distance Difference Test), with the O-GlcNAcylation site (Y89) highlighted. (F) AlphaFold-predicted structure of EWSR1 colored by pLDDT, with the O-GlcNAcylation site (S274) highlighted. Structural features were extracted from AlphaFold predicted structures. pLDDT represents per-residue prediction confidence (0–100). pPSE_24 and pPSE_12 quantify local amino acid packing within 24 Å and 12 Å spheres, respectively, weighted by predicted aligned error (PAE). IDR classification was based on smoothed pPSE_24 values (≤34.27) following the StructureMap methodology.

To identify factors associated with site-level responses, we performed multiple linear regression analysis with standardized coefficients (Figure 5C). Among the structural features examined, the location within the intrinsically disordered region (IDR) was found to be a significant negative predictor of fold change, indicating that O-GlcNAcylation sites in structured regions exhibited larger increases under the N-glycosylation inhibition. This pattern was consistent across all three cell types, with sites in structured regions showing higher average log_2_(Tuni/Ctrl) values compared to those in IDR regions (Figure 5D).

Some examples are shown in Figure 5E to illustrate the differential responses based on the structural context. PRDX6 is a bifunctional cytoplasmic enzyme with both glutathione peroxidase and phospholipase A2 activities that plays critical roles in antioxidant defense and membrane lipid repair.^48^ PRDX6 deficiency induces ER stress and activates the unfolded protein response, and cells lacking PRDX6 exhibit heightened sensitivity to tunicamycin treatment.^49^ The O-GlcNAcylation site at Y89, located within the thioredoxin domain (residues 5–169) in a highly structured region, was upregulated by 2.83-fold under the inhibition of protein N-glycosylation. The thioredoxin domain undergoes conformational changes during catalysis, and the increased O-GlcNAcylation at Y89 may stabilize the thioredoxin conformation and enhance the antioxidant capacity of PRDX6 as part of the cytoprotective response to ER stress.^50^ In contrast, EWSR1 is an RNA-binding protein whose N-terminal low-complexity region modulates phase separation behavior, and it harbors an O-GlcNAcylation site at S274 within its intrinsically disordered region (Figure 5F). This site exhibited minimal change upon the N-glycosylation inhibition (fold change = 0.90), consistent with the observation that O-GlcNAc sites in IDR regions are less responsive to the N-glycosylation inhibition compared to those in structured regions.

### Identification and Quantification of Protein O-GalNAcylation under N-Glycosylation Inhibition

In addition to O-GlcNAcylation, our workflow also allowed us to characterize glycoproteins with the Tn antigen. A total of 485 O-GalNAcylated proteins were identified across the three cell types (Figure 6A and Table S7). GO analysis of all identified O-GalNAcylated proteins revealed enrichment in the cell surface, membrane, extracellular region, transporter activity, glycosaminoglycan binding, and Golgi apparatus (Figure S3A), consistent with the expected subcellular localization of mucin-type O-glycosylated proteins in the classical secretory pathway. Cell-type-specific GO analysis further revealed that HepG2-exclusive O-GalNAcylated proteins were enriched in the extracellular space and ER lumen, whereas Jurkat-exclusive O-GalNAcylated proteins were enriched in glycoprotein metabolism and the Golgi membrane (Figure 6B).

**Figure 6:**
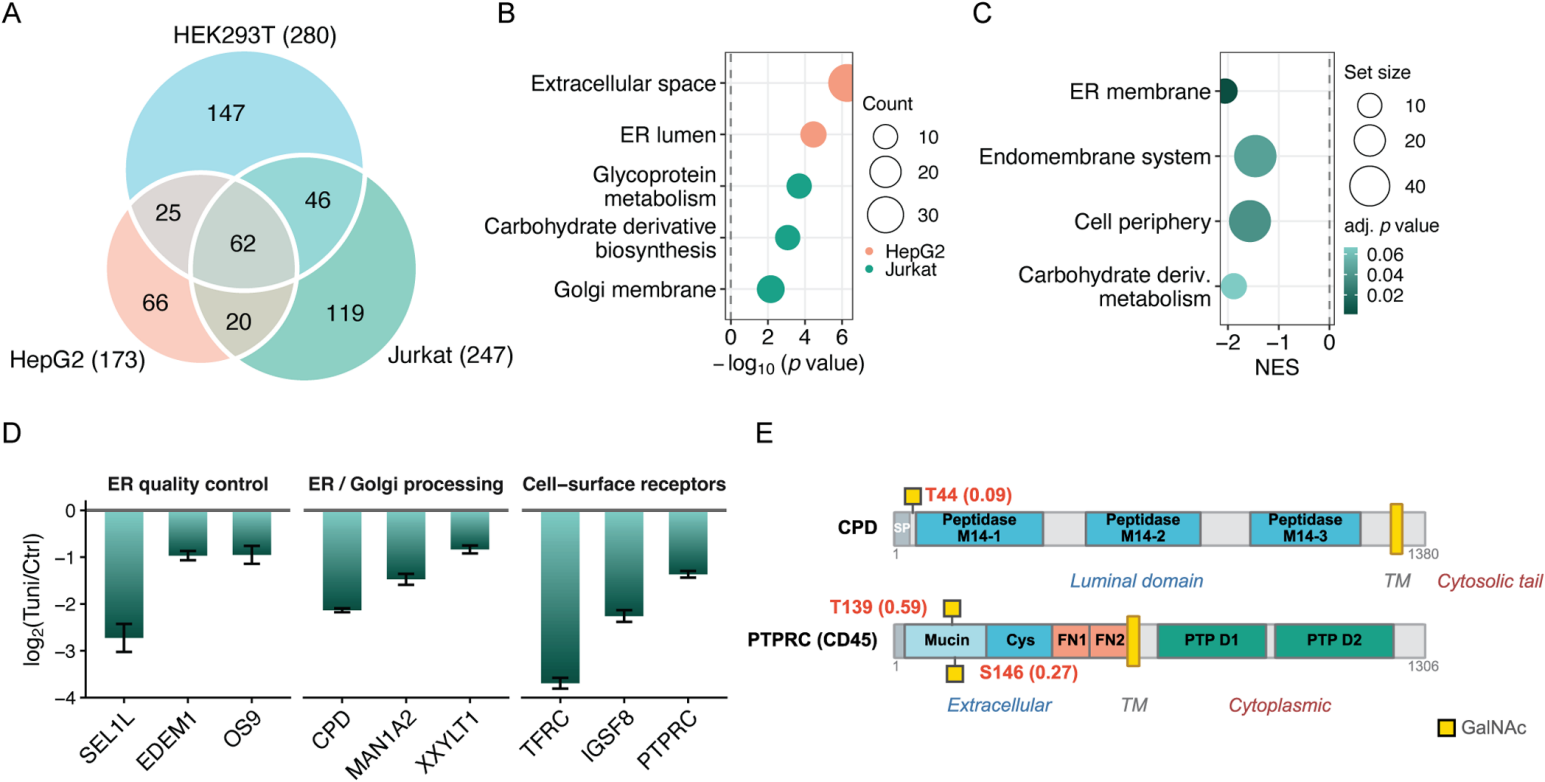
Analysis of protein O-GalNAcylation in HEK293T, HepG2, and Jurkat cells under N-glycosylation inhibition. (A) Overlap of identified O-GalNAcylated proteins across three cell types. (B) GO enrichment analysis of O-GalNAcylated proteins identified exclusively in HepG2 or Jurkat cells. (C) GSEA of O-GalNAcylated proteins in Jurkat cells upon Tm treatment. (D) Log_2_(Tuni/Ctrl) values of selected downregulated O-GalNAcylated proteins in Jurkat cells. (E) Domain architecture and positions of representative downregulated O-GalNAcylation sites in CPD and PTPRC (CD45).

To investigate the abundance changes of protein O-GalNAcylation under the N-glycosylation inhibition, we performed quantitative analysis using the TMT reporter ion intensities from the same multiplexed experiment (Table S8). At the site level, the log_2_(Tuni/Ctrl) distributions differ across cell types (Figure S3B, Table S9 and S10), and the distribution in HEK293T cells showed a significant difference from those of HepG2 and Jurkat cells. These results suggest that the O-GalNAcylation changes under the N-glycosylation inhibition are also cell-type-dependent. The Tn antigen is well-known to be abundantly expressed in Jurkat cells due to the mutation in the *COSMC* gene, which encodes the molecular chaperone required for T-synthase folding.^51–53^ To gain insights into the regulated O-GalNAcylated proteins in Jurkat cells, we performed gene set enrichment analysis (GSEA) (Figure 6C). Notably, ER membrane, endomembrane system, cell periphery, and carbohydrate derivative metabolic process were enriched among the downregulated O-GalNAcylated proteins, indicating that the abundance of O-GalNAcylated proteins in the classical secretory pathway decreased in Jurkat cells under the Tm treatment.

To further characterize the abundance changes of O-GalNAcylated proteins related to the secretory pathway and endomembrane system, we found that these downregulated proteins could be separated into three major groups with different functions, including ER quality-control factors, Golgi/glycan-processing enzymes, and cell-surface receptors (Figure 6D). For example, SEL1L, EDEM1, and OS9, all of which are associated with glycoprotein surveillance and ER-associated degradation (ERAD), were downregulated. EDEM1 delivers misfolded glycoproteins to the SEL1L-containing ERAD complex for retrotranslocation, and OS9 acts as an ER lectin that recognizes mannose-trimmed N-glycans on misfolded substrates, targeting them for degradation.^54–56^ ER/Golgi processing enzymes, such as CPD, MAN1A2, and XXYLT1, were also downregulated. MAN1A2 participates in Golgi mannose trimming during N-glycan processing, XXYLT1 extends O-glucose on EGF repeats and thereby regulates Notch receptor signaling, and CPD is a trans-Golgi carboxypeptidase that cycles between the trans-Golgi network (TGN) and the cell surface to process secretory cargo.^57–62^ Furthermore, cell-surface receptors including TFRC, PTPRC/CD45, and IGSF8, were downregulated, and O-glycosylation can directly influence receptor stability, proteolytic susceptibility, and signaling. For example, the transferrin receptor carries an O-glycan at T104 that protects its juxtamembrane region from proteolytic cleavage.^63,64^ PTPRC/CD45 is a heavily O-glycosylated leukocyte phosphatase whose O-glycans modulate galectin-1 binding, phosphatase signaling, and T-cell death.^65,66^ Overall, the downregulated O-GalNAcylated proteins were involved in ER proteostasis, glycoprotein maturation, and receptor regulation.

We next examined some examples of downregulated O-GalNAcylation sites in the structural context (Figure 6E). For example, for CPD (discussed above), the site of T44 is located at the luminal N-terminus of carboxypeptidase D, immediately downstream of the signal peptide and before the three M14 catalytic domains.^61,62^ A prior study demonstrated that CPD is synthesized as an immature Endo H-sensitive glycoprotein and converted into a more mature Endo H-resistant Golgi/TGN-associated form after exiting from the ER, with the mature form preferentially entering TGN-derived vesicles.^67^ Because N-glycosylation was inhibited by Tm, newly synthesized CPD lacked the N-glycans required for proper folding, leading to its ER retention and reduced export to the Golgi, thereby diminishing the mature Golgi/TGN-accessible CPD pool. This resulted in reduced O-GalNAcylation at T44 being therefore consistent with decreased exposure of this protein to Golgi-resident ppGalNAc transferases under the Tm treatment.

PTPRC/CD45 contained two downregulated sites, T139 and S146, within the N-terminal mucin-like domain of the extracellular disordered region, upstream of the cysteine-rich domain and fibronectin type III repeats. The blocking of N-glycosylation was shown to inhibit the subsequent incorporation of O-linked glycans on mature CD45 and reduced the surface expression of mature CD45 glycoforms.^68,69^ The reduced O-GalNAcylation at T139 and S146 is consistent with impaired O-glycan incorporation on CD45 under Tm treatment. Because CD45 glycosylation modulates T-cell signaling and galectin-mediated apoptotic responses, this reduction may impair CD45-dependent signaling and alter apoptotic responses in Jurkat cells.^66,70^ Together, these examples indicate that decreased O-GalNAcylation occurs in distinct structural contexts in proteins in the secretory pathway from the Golgi to the cell surface.

## Discussion

Protein O-GlcNAcylation and N-glycosylation play critically important roles in many cellular events through modulating protein folding, stability, and interactions with other molecules,^71,72^ and UDP-GlcNAc serves as the sugar donor for both protein O-GlcNAcylation and N-glycosylation, and O-GlcNAcylation of rate-limiting enzymes in the N-glycosylation pathway was reported to impact their functions.^9^ Therefore, it is expected that these two types of protein glycosylation are strongly related. However, systematic and quantitative analysis of the responses of protein O-GlcNAcylation to N-glycosylation inhibition in human cells remains to be explored.

Here, we comprehensively quantified O-GlcNAcylated proteins with the inhibition of N-glycosylation in three types of human cells (HEK293T, HepG2, and Jurkat cells) and investigated its impacts on protein O-GlcNAcylation. By integrating metabolic labeling, bio-orthogonal chemistry, and multiplexed proteomics, we identified and quantified 1,109 O-GlcNAcylated proteins. A methodological consideration in this study is the differentiation between O-GlcNAc and O-GalNAc (the Tn antigen), as Ac_4_GalNAz can be metabolically incorporated into both modification types. To address this challenge, we employed an enzyme, i.e., GAO, which can specifically recognize O-GalNAc but not O-GlcNAc, to treat enriched glycopeptides, followed by methoxylamine labeling to selectively derivatize glycopeptides with O-GalNAc. In this case, it increases the confidence in identifying and quantifying O-GlcNAcylated proteins. This approach provides a clear distinction between these structurally similar but functionally distinct modifications.

For glycopeptide analysis, we implemented an HCD-pd-EThcD fragmentation method combined with O-Pair search for data analysis.^33,73–77^ In this workflow, HCD fragmentation enables efficient glycopeptide identification, while product-dependent EThcD fragmentation preserves the labile O-glycosidic bond and produces c/z ions, allowing for confident localization of O-GlcNAcylation sites. This combined method provides improved confidence in both modification identification and site assignment compared to HCD-only fragmentation.

The current results revealed both common and cell-type-specific changes of O-GlcNAcylated proteins in cells with N-glycosylation inhibition. Among commonly upregulated O-GlcNAcylated proteins, those related to the organophosphate metabolic process, regulation of translation, response to glucose, and positive regulation of RNA splicing were enriched. Among commonly downregulated proteins, GO terms including nuclear protein-containing complex, regulation of response to stress, regulation of translational initiation, and cytoplasmic stress granules were highly overrepresented. Under the inhibition of protein N-glycosylation, O-GlcNAcylated proteins related to the stress response and translation are commonly changed in different types of cells. Besides the common changes across cell types, we also investigated cell-type-specific alterations, and the results demonstrate that the responses of protein O-GlcNAcylation are highly specific to individual cell types. For example, in Jurkat cells, proteins involved in leukocyte proliferation and T-cell activation were upregulated.

A notable finding from this study is the association between protein structural context and site-level response to N-glycosylation inhibition. Multiple linear regression analysis revealed that O-GlcNAcylation sites located within IDRs exhibited significantly smaller abundance changes compared to sites in structured regions. This pattern was consistent across all three cell types, indicating that the structural context is a general determinant of O-GlcNAcylation site changes. These observations suggest that O-GlcNAcylation sites in structured regions may be more responsive to cellular perturbations, potentially because of changes in protein conformation or altered accessibility to OGT under stress conditions. The preferential responsiveness of the O-GlcNAcylation site in structured regions may reflect the exposure of previously buried regions upon protein misfolding induced by the N-glycosylation inhibition, thereby increasing their accessibility for O-GlcNAcylation.

Although O-GlcNAcylation is enriched in IDRs under normal conditions, functional studies have demonstrated that O-GlcNAcylation in structured domains can directly modulate the protein activity. For example, O-GlcNAcylation at S729 within the catalytic domain of EZH2 is required for its di- and trimethylation activity, while the sites in the N-terminal IDR regulate protein stability.^78^ Similarly, O-GlcNAcylation of CaMKII at Ser279 in its regulatory domain confers autonomous kinase activation, and O-GlcNAcylation of AKT at T430/T479 promotes its phosphorylation and downstream signaling transduction.^79,80^ These examples illustrate that O-GlcNAcylation sites in structured regions can serve as direct regulators of enzymatic activity and signaling transduction. The differential responsiveness of O-GlcNAcylated sites in the structural context represents a previously uncharacterized aspect of O-GlcNAcylation dynamics during ER stress. Future studies integrating site-specific functional characterization with structural dynamics will be needed to determine whether this differential responsiveness reflects distinct regulatory roles or simply differences in site accessibility under stress conditions.

## Conclusions

In this work, we systematically investigated protein O-GlcNAcylation changes in different types of human cells under the N-glycosylation inhibition using an integrative method combining metabolic labeling, bio-orthogonal chemistry, and multiplexed proteomics. The results reveal the common and cell-type-specific changes of protein O-GlcNAcylation in cells with the inhibition of protein N-glycosylation. The changes in protein O-GlcNAcylation with N-glycosylation perturbation are related to protein functions. Site-specific analysis further reveals that O-GlcNAcylation sites in structured regions show larger abundance changes compared with those in intrinsically disordered regions. This work provides valuable information regarding the responses of protein O-GlcNAcylation in cells under the N-glycosylation inhibition, advancing our understanding of protein glycosylation in regulating protein functions and cellular events.

## Supplementary Material

























## Data Availability

The MS proteomics data were deposited to the ProteomeXchange Consortium via the PRIDE^81^ partner repository with the data set identifier PXD073249.

## Notes

Experimental details, database search parameters, and bioinformatic analyses and supporting Figures S1–S3 for O-GlcNAcylation quantification reproducibility, GO enrichment, and O-GalNAcylation analysis (PDF)
Identification of O-GlcNAcylated proteins in HEK293T, HepG2, and Jurkat cells (XLSX)
Abundance changes of O-GlcNAcylated proteins in HEK293T, HepG2, and Jurkat cells with the inhibition of N-glycosylation (XLSX)
Identification of total proteins (whole proteome) in HEK293T, HepG2, and Jurkat cells (XLSX)
Abundance changes of total proteins (whole proteome) in HEK293T, HepG2, and Jurkat cells with the inhibition of N-glycosylation (XLSX)
Identification of O-GlcNAcylation sites in HEK293T, HepG2, and Jurkat cells (XLSX)
Abundance changes of O-GlcNAcylation sites in HEK293T, HepG2, and Jurkat cells with the inhibition of N-glycosylation (XLSX)
Identification of O-GalNAcylated proteins in HEK293T, HepG2, and Jurkat cells (XLSX)
Abundance changes of O-GalNAcylated proteins in HEK293T, HepG2, and Jurkat cells with the inhibition of N-glycosylation (XLSX)
Identification of O-GalNAcylation sites in HEK293T, HepG2, and Jurkat cells (XLSX)
Abundance changes of O-GalNAcylation sites in HEK293T, HepG2, and Jurkat cells with the inhibition of N-glycosylation (XLSX)
List of S-GlcNAcylation sites in HEK293T, HepG2, and Jurkat cells (XLSX)
