# Supplementary material for: Systematic Quantification of Protein O‑GlcNAcylation Reveals Common and Cell-Type-Specific Responses to N‑Glycosylation Inhibition in Human Cells
Source: Anal Chem. 2026 May 19;98(21):15689–99. doi: 10.1021/acs.analchem.6c00972 (PMC13234820; doi:10.1021/acs.analchem.6c00972)
Supplement: Supplementary file 1 [file ac6c00972_si_001.pdf]

Supporting Information

Systematic Quantification of Protein O-GlcNAcylation Reveals Common and Cell-Type-Specific Responses to N-Glycosylation Inhibition in Human Cells

Longping Fu, Kejun Yin, Xing Xu and Ronghu Wu*

School of Chemistry and Biochemistry and the Petit Institute for Bioengineering and Bioscience, Georgia Institute of Technology, Atlanta, Georgia 30332, USA

*Corresponding author: Phone: 404-385-1515; Fax: 404-894-7452.

E-mail: ronghu.wu@chemistry.gatech.edu

Supporting Tables

Table S1. Identification of O-GlcNAcylated proteins in HEK293T, HepG2, and Jurkat cells (XLSX)

Table S2. Abundance changes of O-GlcNAcylated proteins in HEK293T, HepG2, and Jurkat cells with the inhibition of N-glycosylation (XLSX)

Table S3. Identification of total proteins (whole proteome) in HEK293T, HepG2, and Jurkat cells (XLSX)

Table S4. Abundance changes of total proteins (whole proteome) in HEK293T, HepG2, and Jurkat cells with the inhibition of N-glycosylation (XLSX)

Table S5. Identification of O-GlcNAcylation sites in HEK293T, HepG2, and Jurkat cells (XLSX)

Table S6. Abundance changes of O-GlcNAcylation sites in HEK293T, HepG2, and Jurkat cells with the inhibition of N-glycosylation (XLSX)

Table S7. Identification of O-GalNAcylated proteins in HEK293T, HepG2, and Jurkat cells (XLSX)

Table S8. Abundance changes of O-GalNAcylated proteins in HEK293T, HepG2, and Jurkat cells with the inhibition of N-glycosylation (XLSX)

Table S9. Identification of O-GalNAcylation sites in HEK293T, HepG2, and Jurkat cells (XLSX)

Table S10. Abundance changes of O-GalNAcylation sites in HEK293T, HepG2, and Jurkat cells with the inhibition of N-glycosylation (XLSX)

Table S11. A list of S-GlcNAcylation sites in HEK293T, HepG2, and Jurkat cells (XLSX)

Experimental Section

Cell lysis and protein digestion

After the treatment, cells were harvested and then washed with ice-cold Dulbecco's phosphate-buffered saline (DPBS, Sigma-Aldrich) twice. They were lysed with a buffer containing 50 mM 4-(2-hydroxyethyl)piperazine-1-ethane-sulfonic acid (HEPES, Sigma-Aldrich), pH 8.6, 150 mM NaCl (Sigma-Aldrich), 1% sodium deoxycholate (SDC, Sigma-Aldrich), 50 μM Thiamet G (TMG, Cayman Chemical), 25 units/mL Benzonase® Nuclease (Sigma-Aldrich), and protease inhibitor cocktail (EDTA-Free, 100X in DMSO, Selleckchem) by tip sonication (Amplitude 35%, 5s on and 5s off, 5 cycles, QSONICA Q125). Then the lysates were centrifuged for 10 min at 4,696 g, and the debris was discarded. The protein concentration was measured using the Pierce™ BCA Protein Assay Kit (Thermo Scientific).

Proteins were reduced with 10 mM dithiothreitol (DTT, Sigma-Aldrich) at 56 °C for 30 min and then were alkylated with 20 mM iodoacetamide (IAA, Sigma-Aldrich) for 30 min in the dark at room temperature. The alkylation reaction was quenched by incubating with 10 mM DTT in the dark for another 15 min. Then, proteins were purified using the chloroform/methanol precipitation method. Purified proteins were digested with sequencing-grade modified trypsin (Promega) in digestion buffer containing 1.6 M urea, 50 mM HEPES, and 50 mM ammonium bicarbonate (pH 8.0) at 37 °C overnight. The digestion was quenched by adding trifluoroacetic acid (TFA, Sigma-Aldrich) until the pH was ~2. Peptides were

purified using a Sep-Pak Vac tC18 cartridge (Waters) and dried in a lyophilizer.

Click chemistry and glycopeptide enrichment

For glycoprotein analysis, labeled glycoproteins were subjected to the copper(I)-catalyzed azide-alkyne cycloaddition (CuAAC) reaction. Briefly, 200 μM photocleavable (PC) Biotin Alkyne (Vector Laboratories), 1 mM $CuSO_4$ (Sigma-Aldrich) and 2 mM 2-(4-((bis((1-(tert-butyl)-1H-1,2,3-triazol-4-yl)methyl)amino)methyl)-1H-1,2,3-triazol-1-yl)acetic acid (BTTAA, Vector Laboratories) were added to the cell lysates. After thorough mixing, freshly prepared 15 mM sodium L-ascorbate (Sigma-Aldrich) was added to initiate the reaction. The reaction was protected from light and lasted for 2 h at room temperature.

For glycopeptide enrichment, purified peptides were enriched using Pierce™ High Capacity NeutrAvidin™ Agarose resins (Thermo Scientific) according to the manufacturer's protocol with some modifications. Briefly, peptides were incubated with the resins at room temperature for 1 h. Then the resins were transferred to a spin column (Thermo Scientific) and washed seven times with 100 mM phosphate buffered saline (PBS) and three times with deionized water (diH_2O). Finally, the resins were resuspended in diH_2O and transferred to a glass vial, and enriched glycopeptides were eluted under the radiation (365 nm) for 25 min at room temperature twice. The eluents were combined, desalted, lyophilized and stored at -80 °C.

TMT labeling and fractionation

Dried glycopeptides from the treatment and control samples were labeled with each channel (Tuni: 126, 127, 128; Ctrl: 129, 130, 131) of the six-plex Tandem Mass Tag (TMT) Isobaric Label Reagent Set (Thermo Scientific), respectively, following the manufacturer's protocol. The labeled samples were mixed, desalted, and lyophilized. Peptides were separated by high-pH HPLC into 12 fractions using a 40-min gradient of 5% to 55% acetonitrile (ACN, Birch Biotech) in 10 mM ammonium acetate (Sigma-Aldrich), pH 10. Each fraction was purified using the stage-tip method and then lyophilized.

GAO oxidation and methoxylamine labeling

The enriched glycopeptides were incubated in 1 mL of oxidation solution containing 25 units of galactose oxidase (GAO, Innovative Research), 40 units of horseradish peroxidase (HRP, Sigma-Aldrich), 500 units of catalase (CAT, Sigma-Aldrich), 20% DMSO, and 25 mM PBS (pH 7.4) at 37 °C for 1 h. The oxidation reaction was quenched by adding $Na_2S_2O_3$ to a final concentration of 1 mM and incubating at room temperature for 1 h. Subsequently, 60.7 μL of methoxylamine hydrochloride solution (Sigma-Aldrich) was added, along with 100 μL of 1 M sodium acetate, 20 μL of aniline, and acetic acid (HOAc) to a final pH 4.5. The methoxylamine labeling reaction proceeded at room temperature overnight. After the reaction, samples were centrifuged at 18,000 × g for 10 min, diluted with 0.05% HOAc to reduce the DMSO

concentration to <2%, and desalted. The resulting glycopeptides were then subjected to high-pH HPLC fractionation.

LC-MS/MS analysis

The samples were dissolved in 6 μL 0.1% formic acid (FA, Sigma-Aldrich), and 4 μL was loaded onto a microcapillary column (75 μm i.d. × 18 cm) packed with C18 beads (ReproSil-Pur 120 C18-AQ, 1.9 μm, Dr. Maisch). Peptides were separated using a Vanquish Neo UHPLC system (Thermo Scientific) at a flow rate of 300 nL/min with a 98 min gradient of buffer A (0.1% FA in water) and buffer B (0.1% FA in ACN): 1% buffer B at 0 min, 8% buffer B at 1 min, 10% buffer B at 30 min, 20% buffer B at 90 min, 99% buffer B at 90.1 min, 99% buffer B at 95 min, 1% buffer B at 96 min, and 1% buffer B at 98 min. Mass spectrometry (MS) analysis was performed with a data-dependent maximum 3s method in an Orbitrap Eclipse Tribrid Mass Spectrometer (Thermo Scientific). Full MS scans were acquired in the Orbitrap at a resolution of 60,000 (at m/z 200), with a scan range of 350-1,500 m/z, standard AGC target, and a maximum injection time of 50 ms. Precursors with charge states 2-8 were selected for fragmentation with a dynamic exclusion duration of 45 s. HCD product-dependent EThcD (i.e., HCD-pd-EThcD) was used for MS/MS acquisition. The initial HCD scan was performed at a normalized collision energy of 38%. EThcD fragmentation was triggered when more than two oxonium ions (m/z 138.0546, 144.0652, 168.0652, 186.0754, 204.0864, 300.1308, 327.1417,

529.2937, and 556.3046) exceeded the threshold of 5% relative intensity. EThcD MS/MS parameters were as follows: quadrupole isolation with an isolation window of 0.7 m/z; ETD reaction time of 30 ms; supplemental activation (SA) collision energy of 35%; Orbitrap detection at a resolution of 15,000 (at m/z 200) with a scan range of 120-4000 m/z; standard normalized AGC target; and maximum injection time of 200 ms.[1, 2]

Database search and data filtering

For glycopeptides analysis, the raw files were converted to the mzML format using MSConvert from ProteoWizard, using peak picking and zero samples removing from the vendor algorithm and keeping the 1,000 most intense peaks.[3] Files were then searched using MSFragger version 4.4 in Fragpipe version 24.0 against the Swiss-Prot Homo sapiens database (20,468 entries). The glyco-O-Pair workflow was used with adjustments based on current experiment design. For modifications, max variable mods on a peptide were set to 4, with carbamidomethylation of cysteine (+57.0214 Da), oxidation of methionine (+15.9949 Da) and protein N-termini acetylation (+42.0106 Da). Mass offsets were set to 0, 299.1230, 326.1339, 528.2859, and 555.2968 Da. Restricted delta masses were applied to serine (S), threonine (T), tyrosine (Y), and cysteine (C) residues. The offset at 299.1230 Da corresponds to the azido-HexNAc modification with the photocleavable biotin remnant after UV cleavage (HexNAz-PC), while 326.1339 Da corresponds to the same modification after GAO oxidation and methoxylamine

labeling (HexNAz-PC-GAO-$MeONH_2$, +27.0109 Da mass shift). The offsets at 528.2859 Da and 555.2968 Da correspond to the TMT-labeled versions of these modifications, respectively. O-Pair glycan database was updated accordingly, and minimum oxonium ion relative abundance is set to 5%. For quantification, TMT reporter ion intensity was extracted by IonQuant and label type was set to TMT-6. The results in psm.tsv file were used for downstream analysis.

Previous studies have demonstrated that per-O-acetylated sugar analogs can induce non-enzymatic S-glycosylation on cysteine residues, which may interfere with O-GlcNAcylation identification.[4] To avoid the interference of S-GlcNAcylation, we applied the following stringent criteria outlined previously.[5, 6] Any glycopeptides with glycan localized on the cysteine residue (confidence level 1 or level 1b) were removed (Table S11). Additionally, any identified glycopeptides with a cysteine residue in their sequences with confidence level 2 or level 3 were also deleted. Eventually, only confidently localized glycopeptides (confidence level 1 or level 1b) with the glycan on the serine, threonine or tyrosine residue were used for site-specific analysis. To further ensure confident site localization, a site probability threshold of ≥0.75 was applied to all glycopeptide-spectrum matches, consistent with established thresholds in O-GlcNAc proteomics studies using HCD-pd-EThcD fragmentation.[7-9]

For whole proteome analysis, the raw files were converted to the mzXML format and searched against the human (*Homo sapiens*) protein database (20435 entries) using SEQUEST (version 28).[10] The following parameters were used during the search: 10 ppm precursor mass

tolerance; 0.025 Da fragment ion tolerance; fully digested with trypsin; up to three missed cleavages; fixed modifications: TMT modification of lysine, the peptide N terminus (+229.1629 Da), and carbamidomethylation of cysteine (+57.0214 Da); variable modifications: oxidation of methionine (+15.9949 Da). The false discovery rates (FDR) of identified peptides and proteins were evaluated by the target-decoy method.[11] Linear discriminant analysis (LDA) was employed to control the quality of peptide identification using multiple parameters including XCorr, ΔCorr, missed cleavage, precursor mass error, peptide length and charge state. The FDRs were controlled to <1%. For quantification, the isotopic information provided by Thermo Scientific was utilized to calibrate the ion intensities. The Signal-to-Noise (S/N) ratio of the TMT reporter ions was used. S/N > 5 is required for quantification of each peptide.

Bioinformatics analysis

All data processing and statistical analysis were performed using RStudio (version 2024.9.0.375) with R (version 4.4.1). Data normalization to minimize technical variation was performed using sample loading (SL) correction followed by the Trimmed Mean of M values (TMM) method implemented in the edgeR package (version 4.2.2). The $\log_2$-transformed intensity ratios of glycopeptides and glycoproteins from three replicates of the tunicamycin treatment and control samples were compared using the limma package (version 3.60.6) with empirical Bayes moderated t-tests. Glycopeptides and glycoproteins exhibiting

$|\log_2(\text{Tuni/Ctrl})| > 0.5$ and Benjamini-Hochberg adjusted P value < 0.05 were considered significantly regulated. Gene Ontology (GO) enrichment analysis was performed using the clusterProfiler package (version 4.12.6) with org.Hs.eg.db for human gene annotation. Protein information was extracted from UniProt. Subcellular localization annotations were obtained from the Human Protein Atlas. Protein sequence properties, including isoelectric point (pI) and hydrophobicity, were calculated for 7-amino acid segments centered on glycosylation sites using the Peptides package. Protein sequences were extracted from UniProt FASTA files using the Biostrings package. Structural features of O-GlcNAc sites were extracted from AlphaFold-predicted structures using StructureMap. Features included predicted local distance difference test (pLDDT) scores, secondary structure assignment, and predicted solvent exposure (pPSE) metrics. Intrinsically disordered regions (IDR) were classified using StructureMap's pPSE-based method: residues with smoothed full-sphere exposure (nAA_24_180_pae_smooth10) ≤ 34.27 were designated as IDR. Protein structure visualization was performed using PyMOL, with structures colored by pLDDT confidence scores according to the standard AlphaFold color scheme. Dimensionality reduction for sample clustering was performed using UMAP implemented in Python (umap-learn package) and accessed through the reticulate R package. Data visualization was performed using ggplot2 with additional packages including ggpubr for statistical annotations, patchwork and cowplot for plot arrangement, circlize and ComplexHeatmap for heatmap visualization, eulerr for proportional Venn diagrams, introdataviz for split violin plots, and rstatix for statistical testing.

Experimental design and statistical rationale

HEK293T, HepG2 and Jurkat cells were used in our experiments because they represent different cell types with unique characteristics that can provide insights into the regulation of O-GlcNAcylation on various biological processes, from general cellular function (HEK293T), active metabolism (HepG2), to immune response (Jurkat). Tm is a potent and commonly used inhibitor for protein N-glycosylation based on the reports in the literature.[12, 13]

All experiments were conducted with three biological replicates, and multiplexed proteomic experiments were performed using the six-plex TMT reagents (6 samples for O-GlcNAcylated proteins and 6 samples for total proteins). Adjusted *P* value (Benjamini-Hochberg) was calculated for each glycopeptide and glycoprotein by limma package, and $|\log_2(\text{Tuni/Ctrl})| > 0.5$ and adjusted P value < 0.05 were set as criteria for identifying significantly changed glycoproteins and glycosylation sites.[14]

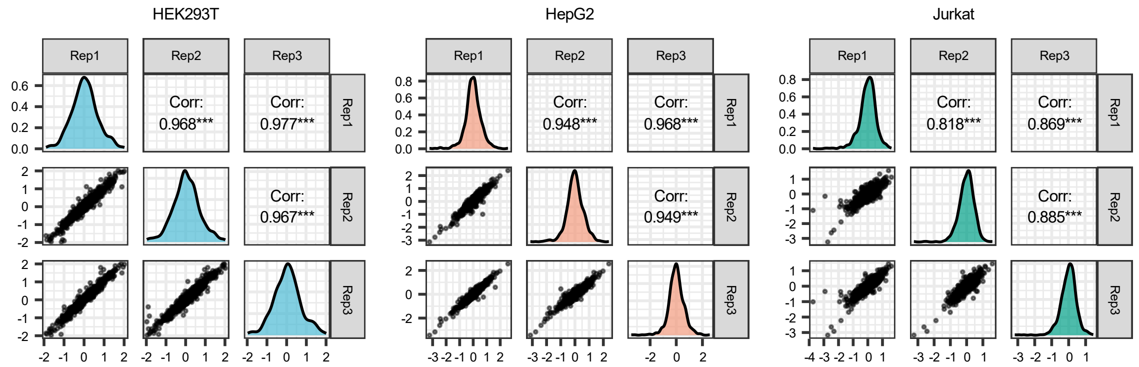

Figure S1. Reproducibility of quantification of O-GlcNAcylated proteins in HEK293T (A), HepG2 (B) and Jurkat (C) cells. Pearson correlation coefficients are shown with significance levels indicated by asterisks (*$p < 0.05$, **$p < 0.01$, and ***$p < 0.001$).

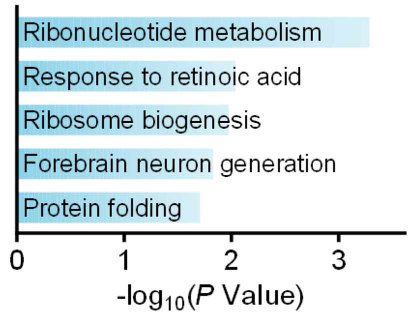

Figure S2. GO terms enriched in glycoproteins upregulated in HEK293T cells.

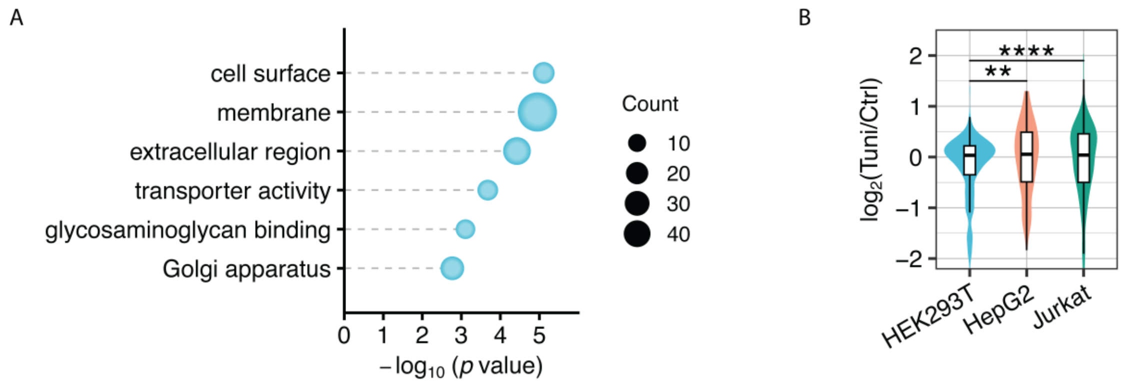

Figure S3. Analysis of protein O-GalNAcylation under the N-glycosylation inhibition. (A) GO enrichment analysis of O-GalNAcylated proteins identified across all cell types. (B) Distributions of fold changes of O-GalNAcylation sites across three cell types.

References

(1) Veth, T. S.; Sutherland, E.; Markuson, K. A.; Zhang, R.; Duboff, A. G.; Huang, J.; Bergen, D.; Lee, A. E.; Melani, R. D.; Canterbury, J. D.; Zabrouskov, V.; McAlister, G. C.; Mullen, C.; Riley, N. M. Improvements in glycoproteomics through architecture changes to the Orbitrap Tribrid MS platform. *Anal. Chem.* **2025**, *97* (22), 11413-11423.

(2) Riley, N. M.; Malaker, S. A.; Driessen, M. D.; Bertozzi, C. R. Optimal dissociation methods differ for N- and O-glycopeptides. *J. Proteome Res.* **2020**, *19* (8), 3286-3301.

(3) Chambers, M. C.*; et al.* A cross-platform toolkit for mass spectrometry and proteomics. *Nat. Biotechnol.* **2012**, *30* (10), 918-920.

(4) Xiao, H.; Wu, R. Global and site-specific analysis revealing unexpected and extensive protein S-GlcNAcylation in human cells. *Anal. Chem.* **2017**, *89* (6), 3656-3663.

(5) Xu, S.; Tong, M.; Suttapitugsakul, S.; Wu, R. Spatial and temporal proteomics reveals the distinct distributions and dynamics of O-GlcNAcylated proteins. *Cell Rep.* **2022**, *39* (11), 110946.

(6) Qin, W.; Qin, K.; Fan, X.; Peng, L.; Hong, W.; Zhu, Y.; Lv, P.; Du, Y.; Huang, R.; Han, M.; Cheng, B.; Liu, Y.; Zhou, W.; Wang, C.; Chen, X. Artificial cysteine S-glycosylation induced by per-O-acetylated unnatural monosaccharides during metabolic glycan labeling. *Angew. Chem. Int. Ed. Engl.* **2018**, *57* (7), 1817-1820.

(7) Hou, C.; Zhang, H.; Deng, J.; Wang, X.; Byers, S.; Levi, M.; Pak, D. T. S.; Moremen, K. W.; Pei, H.; Hart, G. W.; Ma, J. Comprehensive evaluation of cleavable bioorthogonal probes for site-specific O-GlcNAc proteomics. *Mol. Cell. Proteomics* **2025**, *24* (10), 101064.

(8) Hou, C.; Deng, J.; Wu, C.; Zhang, J.; Byers, S.; Moremen, K. W.; Pei, H.; Ma, J. Ultradeep O-GlcNAc proteomics reveals widespread O-GlcNAcylation on tyrosine residues of proteins. *Proc. Natl. Acad. Sci. U. S. A.* **2024**, *121* (47), e2409501121.

(9) Liu, J.; Hao, Y.; Wang, C.; Jin, Y.; Yang, Y.; Gu, J.; Chen, X. An optimized isotopic photocleavable tagging strategy for site-specific and quantitative profiling of protein O-GlcNAcylation in colorectal cancer metastasis. *ACS Chem. Biol.* **2022**, *17* (3), 513-520.

(10) Eng, J. K.; McCormack, A. L.; Yates, J. R. An approach to correlate tandem mass spectral data of peptides with amino acid sequences in a protein database. *J. Am. Soc. Mass Spectrom.* **1994**, *5* (11), 976-989.

(11) Elias, J. E.; Gygi, S. P. Target-decoy search strategy for increased confidence in large-scale protein identifications by mass spectrometry. *Nat. Methods* **2007**, *4* (3), 207-214.

(12) Yin, K.; Tong, M.; Sun, F.; Wu, R. Quantitative structural proteomics unveils the conformational changes of proteins under the endoplasmic reticulum stress. *Anal. Chem.* **2022**, *94* (38), 13250-13260.

(13) Czyz, A.; Brutkowski, W.; Fronk, J.; Duszynski, J.; Zablocki, K. Tunicamycin desensitizes store-operated Ca2+ entry to ATP and mitochondrial potential. *Biochem.*

Biophys. Res. Commun. **2009**, *381* (2), 176-180.

(14) Ritchie, M. E.; Phipson, B.; Wu, D.; Hu, Y.; Law, C. W.; Shi, W.; Smyth, G. K. limma powers differential expression analyses for RNA-sequencing and microarray studies. *Nucleic Acids Res.* **2015**, *43* (7), e47.
